# Acute bronchodilator responsiveness and health outcomes in COPD patients in the UPLIFT trial

**DOI:** 10.1186/1465-9921-12-6

**Published:** 2011-01-11

**Authors:** Nicola A Hanania, Amir Sharafkhaneh, Bartolome Celli, Marc Decramer, Ted Lystig, Steven Kesten, Donald Tashkin

**Affiliations:** 1 Section of Pulmonary, Critical Care and Sleep Medicine, Baylor College of Medicine, Houston, TX, USA; 2 Section of Pulmonary, Critical Care and Sleep Medicine, Medical Care Line, Michael E. DeBakey VA Medical Center, Houston, TX, USA; 3 Brigham and Women's Hospital, Boston, Boston, MA, USA; 4 University of Leuven, Leuven, Belgium; 5 Boehringer Ingelheim Pharmaceuticals, Inc, Ridgefield, CT, USA; 6 David Geffen School of Medicine UCLA, Los Angeles, CA, USA

## Abstract

**Background:**

Debate continues as to whether acute bronchodilator responsiveness (BDR) predicts long-term outcomes in COPD. Furthermore, there is no consensus on a threshold for BDR.

**Methods:**

At baseline and during the 4-year Understanding Potential Long-term Improvements in Function with Tiotropium (UPLIFT^®^) trial, patients had spirometry performed before and after administration of ipratropium bromide 80 mcg and albuterol 400 mcg. Patients were split according to three BDR thresholds: ≥12% + ≥200 mL above baseline (criterion A), ≥15% above baseline (criterion B); and ≥10% absolute increase in percent predicted FEV_1 _values (criterion C). Several outcomes (pre-dose spirometry, exacerbations, St. George's Respiratory Questionnaire [SGRQ] total score) were assessed according to presence or absence of BDR in the treatment groups.

**Results:**

5783 of 5993 randomized patients had evaluable pre- and post-bronchodilator spirometry at baseline. Mean age (SD) was 64 (8) years, with 75% men, mean post-bronchodilator FEV_1 _1.33 ± 0.44 L (47.6 ± 12.7% predicted) and 30% current smokers. At baseline, 52%, 66%, and 39% of patients had acute BDR using criterion A, B, and C, respectively. The presence of BDR was variable at follow-up visits. Statistically significant improvements in spirometry and health outcomes occurred with tiotropium regardless of the baseline BDR or criterion used.

**Conclusions:**

A large proportion of COPD patients demonstrate significant acute BDR. BDR in these patients is variable over time and differs according to the criterion used. BDR status at baseline does not predict long-term response to tiotropium. Assessment of acute BDR should not be used as a decision-making tool when prescribing tiotropium to patients with COPD.

## Background

Chronic obstructive pulmonary disease (COPD) is characterized by airflow limitation which is not fully reversible [1]. There has been much interest in whether acute bronchodilator responsiveness (BDR) based on a predefined threshold of a change in forced expiratory volume in the first second (FEV_1_) is a prognostic factor in COPD [2,3]. A 1-year trial with tiotropium showed that acute responsiveness was not predictive of whether patients improved clinically [4]. However, whether such responsiveness can predict disease progression or health outcomes beyond 1 year has not been established. Further, the reliability of currently recommended criteria for assessing responsiveness (also referred to as reversibility) differs according to various guidelines. The American Thoracic Society considers a 200 mL and 12% increase from pre-bronchodilator baseline FEV_1 _as a positive BDR [5], while a 15% increase from baseline [6] or 10% increase in normalized FEV_1 _is considered positive BDR by other groups [2,7,8].

The Understanding Potential Long-term Improvements in Function with Tiotropium (UPLIFT^®^) trial was a 4-year placebo-controlled clinical trial evaluating the long-term effects of tiotropium 18 mcg daily on lung function, exacerbations, health-related quality of life and mortality in a large group of patients with COPD [9,10]. Spirometry was performed before and after administration of short-acting bronchodilators (ipratropium bromide and albuterol) at baseline and during follow-up visits every 6 months throughout the 4-year duration of the study [11]. In addition to lung function data, the study collected information on health-related quality of life, COPD exacerbations and mortality. Therefore, the UPLIFT study provided a unique opportunity to examine several aspects of the BDR in a large cohort of patients with COPD. Data describing the acute bronchodilator response at baseline from the UPLIFT trial were previously published (11). In this study, we further evaluate the acute bronchodilator response over the four years of the trial.

## Methods

### Study design

The UPLIFT study details have previously been reported and are briefly summarized in the subsequent paragraphs [9,10]. The present study was performed to examine: a) the prevalence of significant acute BDR using three predefined criteria in a large cohort of COPD; b) the predictive ability of baseline BDR on lung function and health outcomes over four years, and c) the variability of acute BDR over four years.

### Patients

Patients were recruited from 490 investigational sites in 37 countries. They were eligible for inclusion if they had a diagnosis of COPD, were aged ≥40 years with a smoking history of at least 10 pack-years, had post-bronchodilator FEV_1 _≤70% of predicted, and FEV_1 _to forced vital capacity (FVC) ratio of <0.70. Patients were excluded from participating if they had history of asthma, COPD exacerbation, or respiratory infection within 4 weeks of screening, prior pulmonary resection, were using supplemental oxygen for >12 hours per day, or had significant disease other than COPD that might influence the study results or ability to participate. Patients were permitted to use all respiratory medications (excluding other inhaled anticholinergics) throughout the trial.

### Assessments

Pre- and post-bronchodilator spirometry was performed prior to and after four inhalations of ipratropium (total = 80 mcg) followed 60 minutes later by four inhalations of albuterol (total = 400 mcg). Post-bronchodilator spirometry was performed 30 minutes after inhalation of albuterol. At clinic visits following randomization, study drug was administered immediately prior to administration of short-acting bronchodilators. Medication washout requirements included withholding short- and long-acting β-agonists (for ≥8 and ≥12 hours, respectively), short- and long-acting theophylline preparations (for ≥24 and ≥48 hours, respectively) and antileukotrienes (for ≥48 hours), prior to spirometry. Patients were discouraged from smoking during the study visit and were not permitted to smoke within 30 minutes of spirometry. Patients' self-report was relied upon regarding their adherence to these restrictions, as is routinely the case in clinical trials.

Spirometry and the St. George's Respiratory Questionnaire (SGRQ) [12] were performed every 6 months throughout the trial. Additionally, spirometry was performed 30 days after randomization and requested 30 days after the last dose of study medication. Information on exacerbations, exacerbations leading to hospitalization, and adverse events was collected at all clinic visits. Mortality was analyzed based on fatal events occurring during treatment until 1470 days from randomization.

### Statistical methods

Data from all randomized patients with acceptable pre- and post-bronchodilator measurements at baseline were included in this analysis. Patients were split according to initial FEV_1 _response to short-acting inhaled bronchodilators as previously described, based on three standard criteria: ≥12% and ≥200 mL improvement over baseline (referred to as criterion A); ≥15% increase over baseline (referred to as criterion B); and ≥10 unit (%) absolute increase in the percent predicted value (referred to as criterion C). Changes in FEV_1_, FVC, and SGRQ total score were analyzed using a mixed models repeated measurements (MMRM) analysis of variance approach, which included adjustment for baseline measurement values. Numbers of exacerbations were estimated using Poisson regression, with adjustment for overdispersion and treatment exposure. For decline in lung function, data sets were restricted to patients with at least three post-randomization spirometry test sets. Cox regression was used to calculate hazard ratios for analyses of time to first exacerbation and for mortality.

## Results

### Study population

Baseline demographic data for the full UPLIFT^® ^cohort have been previously reported [10]. A total of 5992 patients were randomized and received study medication in the UPLIFT^® ^study. Of these patients, 5783 patients had bronchodilator responsiveness data at baseline, allowing them to be included in the present analysis. The mean age (SD) was 64 (8) years with 75% being male and 30% being current smokers. Mean (SD) pre-bronchodilator FEV_1 _was 1.10 (0.40) L (39.4 [12.0]% predicted). Mean post-bronchodilator FEV_1 _(SD) was 1.33 (0.44) L (47.6 [12.7]% predicted). Patients' demographics and baseline characteristics were similar when classified according to BDR criteria A, B, and C except for baseline SGRQ total score, which indicated worse health-related quality of life for nonresponders for patients meeting criteria A and C (Table 1). Pre-bronchodilator FEV_1 _was highest in criterion B nonresponders and lowest in the corresponding responders (Table 2).

**Table 1:** Baseline characteristics of tiotropium and placebo groups according to different threshold criteria for bronchodilator responsiveness.

	**Criterion A (∆ % Predicted FEV**_**1 **_**≥12% and ≥200 mL)**				**Criterion B (∆ % Predicted FEV**_**1 **_**≥15%)**				**Criterion C (∆ % Predicted FEV**_**1 **_**≥10%) **			
	Nonresponder (n = 2750)		Responder (n = 3033)		Nonresponder (n = 1995)		Responder (n = 3788)		Nonresponder (n = 3553)		Responder (n = 2230)	
	Tio (n = 1357)	Placebo (n = 1393)	Tio (n = 1520)	Placebo (n = 1513)	Tio (n = 995)	Placebo (n = 1000)	Tio (n = 1882)	Placebo (n = 1906)	Tio (n = 1769)	Placebo (n = 1784)	Tio (n = 1108)	Placebo(n = 1122)
Age (years)	64.9 ± 8.4	65.3 ± 8.4	64.1 ± 8.4	63.8 ± 8.5	64.4 ± 8.3	64.5 ± 8.5	64.5 ± 8.5	64.5 ± 8.5	64.4 ± 8.3	64.6 ± 8.4	64.7 ± 8.6	64.3 ± 8.7
Male, %	69.7	69.2	80.2	78.6	77.0	75.7	74.3	73.2	76.2	75.6	73.7	71.7
Smoking history (%)												
Ex-smoker	69.6	70.7	71.6	69.3	67.8	67.5	72.1	71.2	69.3	70.7	72.727.3	68.8
Current smoker	30.4	29.3	28.4	30.7	32.2	32.5	27.9	28.8	30.7	29.3		31.2
Mean COPD duration (years)	10.1 ± 7.7	9.8 ± 7.7	9.7 ± 7.5	9.6 ± 7.0	9.9 ± 7.6	9.7 ± 7.5	9.9 ± 7.5	9.7 ± 7.3	10.1 ± 7.7	9.7 ± 7.5	9.6 ± 7.4	9.6 ± 7.1
Baseline medication* use (%)												
LABA	60.0	61.5	59.7	59.1	56.9	59.1	61.4	60.8	59.8	61.7	59.9	57.8
ICS	62.1	62.5	60.8	60.7	58.9	59.9	62.8	62.4	61.6	61.7	61.2	61.4
Combination ICS+LABA	49.2	50.0	48.4	47.3	45.2	47.6	50.6	49.1	48.5	49.7	49.1	46.7
Anticholinergic	46.9	47.6	44.9	42.7	44.0	43.9	46.8	45.6	47.1	45.8	43.9	43.9
Theophyllines	31.6	33.0	26.3	24.3	29.0	31.4	28.7	27.0	30.7	31.7	25.8	23.4
SGRQ total score (units)	47.2 ± 17.2	48.0 ± 17.5	44.5 ± 16.7	43.9 ± 16.7	45.4 ± 17.5	46.6 ± 17.8	46.0 ± 16.7	45.5 ± 16.9	46.9 ± 17.2	47.1 ± 17.2	44.0 ± 16.6	43.9 ± 17.0

Data expressed as either proportions or mean ± SD.

Tio = tiotropium; LABA = long-acting β-acting agonist; ICS = inhaled corticosteroid; SGRQ = St. George's Respiratory Questionnaire; GOLD = Global Initiative for Chronic Obstructive Lung Disease.

*baseline maintenance inhaled respiratory medication.

**Table 2:** Mean baseline spirometry according to bronchodilator responsiveness status.

	**Criterion A (∆ % Predicted FEV**_**1 **_**≥12% and ≥200 mL)**				**Criterion B (∆ % Predicted FEV**_**1 **_**≥15%)**				**Criterion C (∆ % Predicted FEV**_**1 **_**≥10%) **			
	Nonresponder (n = 2750)		Responder (n = 3033)		Nonresponder (n = 1995)		Responder (n = 3788)		Nonresponder (n = 3553)		Responder (n = 2230)	
	Tio (n = 1357)	Placebo (n = 1393)	Tio (n = 1520)	Placebo (n = 1513)	Tio (n = 995)	Placebo (n = 1000)	Tio (n = 1882)	Placebo (n = 1906)	Tio (n = 1769)	Placebo (n = 1784)	Tio (n = 1108)	Placebo (n = 1122)
Prebronchodilator												
FEV_1 _(L)	1.09 ± 0.45	1.07 ± 0.43	1.11 ± 0.36	1.11 ± 0.37	1.29 ± 0.44	1.25 ± 0.42	1.00 ± 0.34	1.01 ± 0.36	1.12 ± 0.43	1.10 ± 0.43	1.08 ± 0.35	1.07 ± 0.36
FEV_1 _% predicted	40.7 ± 13.4	40.0 ± 13.2	38.5 ± 10.6	38.6 ± 10.5	45.6 ± 12.2	44.8 ± 12.3	36.3 ± 10.6	36.3 ± 10.6	39.9 ± 13.1	39.6 ± 13.0	38.9 ± 10.1	38.8 ± 10.0
FVC (L)	2.57 ± 0.86	2.54 ± 0.85	2.69 ± 0.76	2.71 ± 0.80	2.84 ± 0.85	2.81 ± 0.84	2.52 ± 0.76	2.53 ± 0.81	2.65 ± 0.83	2.63 ± 0.84	2.59 ± 0.76	2.61 ± 0.81
Postbronchodilator												
FEV_1 _(L)	1.19 ± 0.44	1.16 ± 0.43	1.46 ± 0.39	1.46 ± 0.40	1.36 ± 0.47	1.33 ± 0.45	1.31 ± 0.42	1.32 ± 0.44	1.25 ± 0.44	1.23 ± 0.44	1.46 ± 0.40	1.46 ± 0.41
FEV_1 _% predicted	44.4 ± 13.3	43.7 ± 13.1	50.8 ± 11.3	50.9 ± 11.1	48.3 ± 13.0	47.5 ± 13.1	47.5 ± 12.6	47.4 ± 12.4	44.6 ± 12.9	44.2 ± 12.9	52.9 ± 10.5	52.6 ± 10.3
% ∆ FEV_1_	10.9 ± 10.2	11.1 ± 10.4	34.6 ± 16.0	34.7 ± 15.9	6.14 ± 6.65	6.16 ± 6.82	32.6 ± 15.2	32.4 ± 15.2	13.8 ± 11.2	13.8 ± 11.5	38.7 ± 16.1	38.6 ± 15.7
FVC (L)	2.81 ± 0.84	2.79 ± 0.85	3.36 ± 0.80	3.38 ± 0.86	3.02 ± 0.87	3.00 ± 0.88	3.14 ± 0.86	3.15 ± 0.91	2.97 ± 0.85	2.95 ± 0.87	3.30 ± 0.85	3.33 ± 0.90
GOLD Stage (%)												
II	37.4	34.2	55.9	56.9	49.8	46.6	45.7	45.7	37.0	35.8	63.4	62.2
III	48.0	50.5	41.2	39.7	41.1	43.3	46.1	45.6	50.1	50.2	35.2	36.4
IV	14.7	15.4	2.8	3.4	9.0	10.1	8.0	8.6	12.9	14.0	1.2	1.3

Tio = tiotropium; FEV_1 _= forced expiratory volume in 1 second; FVC = forced vital capacity; GOLD = Global Initiative for Chronic Obstructive Lung Disease.

### Bronchodilator responsiveness

**Figure 1:**
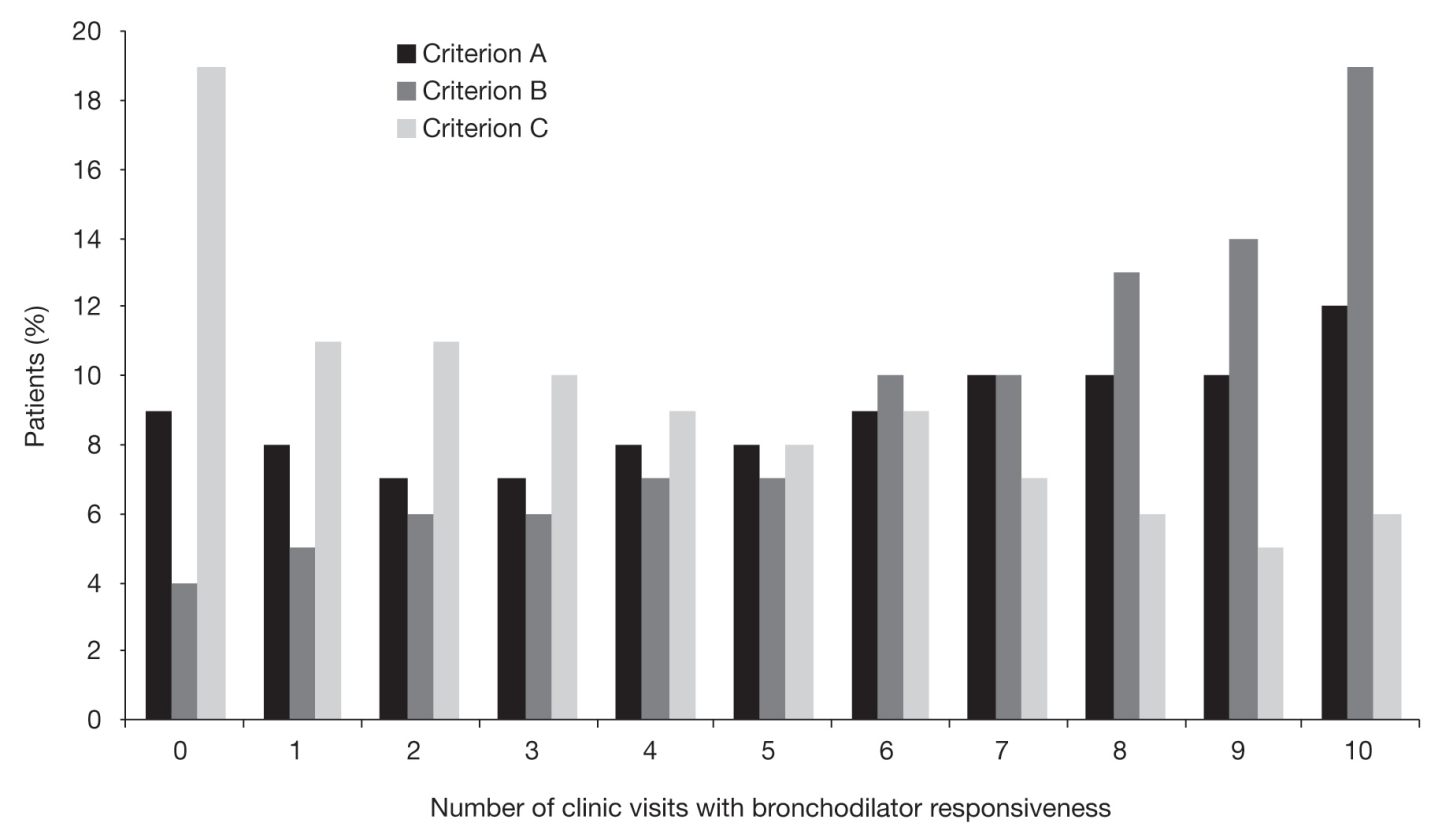
**Proportion of patients who demonstrated bronchodilator responsiveness by number of clinic visits according to criteria A, B, and C**. The histograms on the most left (0) reflect percent of patients who never met the criteria for responsiveness while the ones on the most right (10) reflect the subjects who on all the 10 occasions of testing met the reversibility criteria. Population restricted to the patients randomized to placebo group and who had spirometry performed at all 10 spirometry clinic visits.

A total of 52%, 66%, and 39% of patients exceeded the thresholds for responsiveness defined by criteria A, B, and C at baseline, respectively (Table 3). The percent of patients labeled as responsive diminished with increasing Global Initiative for Chronic Obstructive Lung Disease (GOLD) stage of severity only when criterion A or C was used (Table 3). Figure 1 demonstrates the frequency distribution of BDR with repeated testing using the three criteria in patients randomized to the placebo arm and in whom spirometry was performed at every visit. Analysis of frequency of BDR was restricted to the 1411 patients with full data in the placebo group as bronchodilation due to tiotropium exceeds 24 hours and a full washout prior to clinic visits was not appropriate for the study. A minority of these patients failed to show BDR at any clinic visit (9% for criterion A, 4% for criterion B, and 19% for criterion C). In contrast, some of these patients demonstrated BDR at every clinic visit (12% for criterion A, 19% for criterion B, and 6% for criterion C). Approximately 60%, 73%, and 40% of patients who completed all visits in the placebo group were considered to have BDR on ≥50% of visits according to criteria A, B, and C, respectively.

**Table 3:** Proportion of patients with baseline bronchodilator responsiveness according to GOLD severity stage.

GOLD Stage	n	Criterion A	Criterion B	Criterion C
**II**	2694	1714 (64%)	1735 (64%)	1404 (52%)
**III**	2580	1226 (48%)	1738 (64%)	798 (31%)
**IV**	506	93 (18%)	315 (62%)	28 (6%)
**All**	5783	3033 (52%)	3788 (66%)	2230 (39%)

GOLD = Global Initiative for Chronic Obstructive Lung Disease

### Lung function

The mean annualized rate of decline in FEV_1 _was similar among the different threshold criteria and was not influenced by treatment assignment (Table 4). Pre- and post-bronchodilator lung function (FEV_1_, FVC) improved significantly with tiotropium (p < 0.001 versus placebo for all comparisons), irrespective of whether there was a positive baseline BDR using any of the criteria (Table 4). Furthermore, the degree of improvement in FEV_1 _and FVC was similar or greater in responsive compared to poorly responsive patients.

**Table 4:** Spirometry outcomes in tiotropium and placebo groups according to different threshold criteria for bronchodilator responsiveness.^†^

A	**Criterion A (∆ % Predicted FEV**_**1 **_**≥12% and ≥200 mL)**				**Criterion B (∆ % Predicted FEV**_**1 **_**≥15%)**				**Criterion C (∆ % Predicted FEV**_**1 **_**≥10%)**			
	Nonresponder		Responder		Nonresponder		Responder		Nonresponder		Responder	
Prebronchodilator												
Difference in FEV_1 _at 4 years (mL)*	76 (54, 98)		98 (77, 119)		97 (69, 124)		83 (65, 101)		78 (58, 97)		105 (81, 130)	
Difference in FVC at 4 years (mL)*	134 (89, 178)		195 (153, 236)		143 (92, 194)		179 (140, 217)		139 (99, 179)		213 (165, 260)	

**B**	**Criterion A (∆ % Predicted FEV**_**1 **_**≥12% and ≥200 mL)**				**Criterion B (∆ % Predicted FEV**_**1 **_**≥15%)**				**Criterion C (∆ % Predicted FEV**_**1 **_**≥10%)**			
	**Nonresponder**		**Responder**		**Nonresponder**		**Responder**		**Nonresponder**		**Responder**	
	**Tio**	**Placebo**	**Tio**	**Placebo**	**Tio**	**Placebo**	**Tio**	**Placebo**	**Tio**	**Placebo**	**Tio**	**Placebo**
Rate of change in FEV_1 _(mL/year)^†^												
Prebronchodilator	-32 ± 2	-31 ± 2	-29 ± 2	-29 ± 2	-35 ± 2	-38 ± 2	-28 ± 2	-26 ± 2	-32 ± 2	-32 ± 2	-28 ± 2	-28 ± 2
Postbronchodilator	-37 ± 2	-37 ± 2	-43 ± 2	-47 ± 2	-38 ± 2	-42 ± 2	-42 ± 2	-43 ± 2	-39 ± 2	-39 ± 2	-43 ± 2	-47 ± 2

(A) mean differences (95%CI) at 4 years (tiotropium - placebo), (B) mean (SE) rate of change by treatment group.

^†^In patients with at least three measurements after Day 30. Change in FEV_1 _and FVC data are based on repeated-measures ANOVA model, adjusted for baseline. Rates of decline in FEV_1 _data are based on random-effects model.

*p ≤ 0.001 versus placebo. Tio = tiotropium; FEV_1 _= forced expiratory volume in 1 second; FVC = forced vital capacity.

### Health-related quality of life

**Figure 2:**
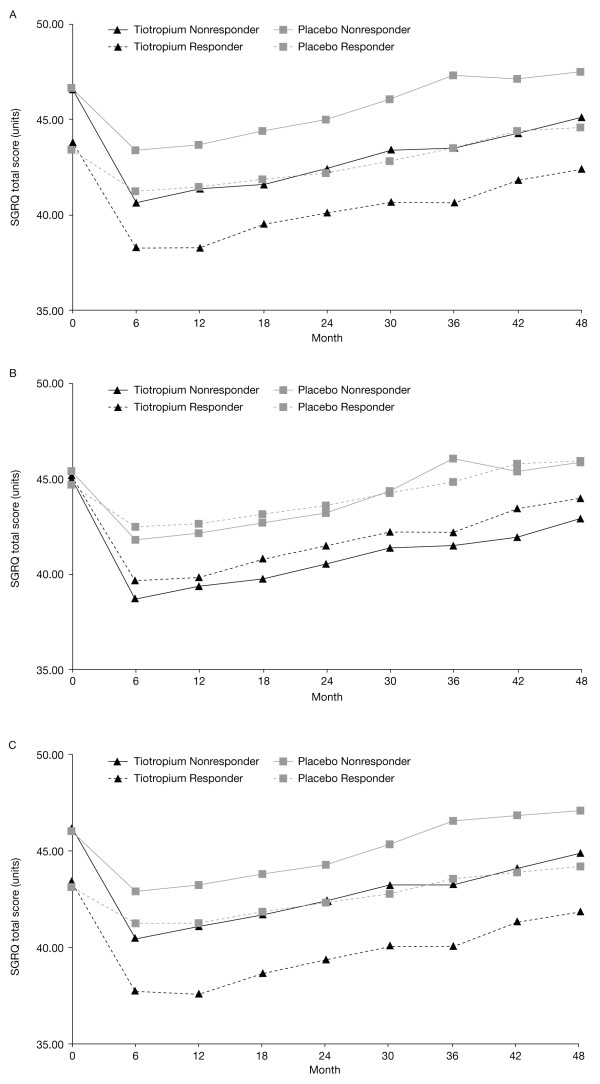
**Mean SGRQ total scores in the tiotropium and the placebo groups according to bronchodilator responsiveness at baseline using criteria A, B, and C**.

Baseline SGRQ were similar between the responsive and poorly responsive patients regardless of the criteria of BDR used (Table 1). Differences in SGRQ total score indicated statistically significant improvements with tiotropium versus placebo in both responsive and poorly responsive groups, regardless of criterion used (Figure 2, p < 0.001 for all comparisons).

### COPD exacerbations

Risk of an exacerbation over the entire trial was reduced with tiotropium in both the responsive and poorly responsive groups at baseline regardless of the BDR criteria. Furthermore, in poorly responsive patients at baseline, tiotropium was associated with significantly fewer exacerbations compared with placebo (p < 0.005, Table 5). The criterion used did not appear to influence the risk or mean number of exacerbations during the trial.

**Table 5:** Exacerbations outcomes in tiotropium and placebo groups according to different threshold criteria for bronchodilator responsiveness.

	Tiotropium	Placebo	Hazard (or Rate) Ratio (95%CI) (tiotropium/placebo)	p-value
**Patients with **≥**1 exacerbation, n (%)**				
Responsiveness Criterion A				
Nonresponder	913 (67.3)	946 (67.9)	0.86 (0.79, 0.94)	0.0014
Responder	1021 (67.2)	1036 (68.5)	0.86 (0.79, 0.94)	0.0008
Responsiveness Criterion B				
Nonresponder	634 (63.7)	676 (67.6)	0.85 (0.76, 0.95)	0.0029
Responder	1300 (69.1)	1306 (68.5)	0.87 (0.80, 0.94)	0.0003
Responsiveness Criterion C				
Nonresponder	1198 (67.7)	1204 (67.5)	0.89 (0.82, 0.96)	0.0036
Responder	736 (66.4)	778 (69.3)	0.82 (0.74, 0.91)	0.0002
**Number of exacerbations/patient year, mean**				
Responsiveness Criterion A				
Nonresponder	0.76	0.87	0.87 (0.79, 0.95)	0.0019
Responder	0.69	0.82	0.85 (0.78, 0.92)	0.0002
Responsiveness Criterion B				
Nonresponder	0.65	0.77	0.84 (0.75, 0.94)	0.0026
Responder	0.76	0.88	0.86 (0.80, 0.93)	0.0001
Responsiveness Criterion C				
Nonresponder	0.74	0.86	0.86 (0.79, 0.93)	0.0001
Responder	0.70	0.81	0.86 (0.77, 0.95)	0.0025

p-value and hazard ratio based on Cox-regression for analysis of time to first exacerbation. p-value and rate ratio based on Poisson-regression with adjustment for overdispersion for analysis of number of exacerbations per patient year.

### All-cause mortality

The hazard ratio for a fatal event (tiotropium relative to the placebo group) was similar in responsive and poorly responsive patients regardless of criteria used. However, poorly responsive patients showed a tendency to higher all-cause mortality regardless of the responsiveness definition used (Table 6).

**Table 6:** All-cause mortality in tiotropium and placebo groups according to different threshold criteria for bronchodilator responsiveness.

	Tiotropium	Placebo	Hazard Ratio (95% CI) (tiotropium/placebo)	p-value
Responsiveness Criterion A				
Nonresponder (%)	208/1357 (15.3)	225/1393 (16.2)	0.84 (0.70, 1.02)	0.07
Responder (%)	152/1520 (10.0)	164/1513 (10.8)	0.86 (0.69, 1.07)	0.17
Responsiveness Criterion B				
Nonresponder	141/995 (14.2)	151/1000 (15.1)	0.87 (0.69, 1.10)	0.24
Responder	219/1882 (11.6)	238/1906 (12.5)	0.84 (0.70, 1.01)	0.06
Responsiveness Criterion C				
Nonresponder (%)	265/1769 (15.0)	283/1784 (15.9)	0.85 (0.72, 1.00)	0.05
Responder (%)	95/1108 (8.6)	106/1122 (9.4)	0.84 (0.64, 1.11)	0.22

## Discussion

Data from the UPLIFT trial demonstrated that the presence or absence of achieving a pre-defined threshold for increases in FEV_1 _after single occasion administration of maximal doses of short-acting bronchodilators in COPD patients (BDR) does not influence whether or not patients will attain long-term improvements in lung function and health-related quality of life along with a reduced risk for exacerbations with tiotropium treatment. Furthermore, the absence of acute BDR at one occasion does not preclude demonstration of BDR on another occasion. Only a small minority (<20%) of the placebo patients who were tested at every visit throughout the trial remained nonresponsive (i.e. did not increase beyond a pre-defined threshold for responsiveness). Finally, the proportion of patients who have BDR was somewhat dependent on the threshold used to define responsiveness.

Bronchodilator responsiveness testing is routinely performed in clinical practice and research studies in patients with COPD. Response to tiotropium compared to placebo was not affected by responsiveness to short-acting bronchodilators at baseline regardless of the definition used and supports findings from an earlier publication of a 1-year trial [4]. The observations have important implications in pharmacotherapy of stable COPD and suggest that assessment of acute BDR using a predefined threshold should not be used as a decision-making tool when prescribing tiotropium for patients with COPD. The data and conclusions also confirm previous reports with other COPD treatments [13,14].

COPD is defined as a disease characterized by partially reversible airflow limitation. Responsiveness (or reversibility) criteria vary among various professional societies. Advantages and disadvantages of using any of the proposed criteria have been extensively discussed in the literature. To summarize, published reports suggest that a 12 to 15% increase in FEV_1 _compared to baseline exceeds normal within-subject variability and response to placebo inhalation [15,16]. However, a low baseline FEV_1 _may produce a high percent improvement from baseline with only a small absolute improvement. Thus using an absolute volume increase has been considered relevant. A threshold of 200 mL has traditionally been used, although this stems from the asthma literature since a change of 100 to 150 mL in FEV_1 _in COPD is usually considered clinically significant as it exceeds the minimal clinical significant difference [17]. Additional data from Herpel *et al*. support the use of minimal absolute volume change as a criterion [18]. An additional consideration is the use of lung volumes such as inspiratory capacity and FVC in response to bronchodilators in COPD as volume changes may be more pronounced and may correlate more with clinical outcomes than a change in FEV_1 _[19,21].

A unique characteristic of the UPLIFT data is the repeated spirometry with acute bronchodilator testing over 10 sessions in 4 years. Upon examining the data from patients in the placebo group who had spirometry at every visit, it is apparent that there is a wide variability in the occurrence of BDR with serial spirometry, which is consistent with data previously described in other studies [22]. Among the three criteria used, criterion B classified the highest percentage of patients as always responsive, while criterion C identified highest percentages of patients as always nonresponsive. Further, as severity of COPD increased, the percent of responsive patients decreased when criterion A or C was used, while it did not change appreciably with criterion B. The differences among the thresholds again highlight the influence of inclusion of absolute volume changes. However, the more important finding is the confirmation that measurement of BDR varies with time and a one-time measurement has limited importance in the management of COPD patients.

The predictive value of achieving BDR based on predefined thresholds as a marker distinguishing patients who will have long-term positive outcomes with pharmacotherapy in COPD has been a matter of debate. Bronchodilator responsiveness grouping according to various definitions may be associated with somewhat different magnitudes of responses but the results presented here from the 4-year UPLIFT trial confirm and extend findings from previous studies that the absence of BDR does not preclude a long-term clinical response [4,13,14]. This suggests that the predictive ability of BDR testing using a pre-determined threshold is limited.

The present study demonstrated that all-cause mortality tends to be lower in responsive patients than those who did not have any BDR at baseline. This difference was most pronounced when criterion A or C was used (Table 5). This is in contrast to data from Hansen and colleagues who studied a large cohort of patients with COPD followed for an average of 11.2 years and showed that bronchodilator responsiveness did not predict mortality when the best post-bronchodilator baseline FEV_1 _was used in the model [23].

In addition to the criterion of BDR used, other factors may influence the presence or absence of BDR when spirometry is performed. One such factor includes the dose and type of short-acting bronchodilator used to test BDR. Traditionally, two to four inhalations of albuterol are used with post-bronchodilator testing performed 10 to 20 minutes post-treatment. However, some patients with COPD may not respond to albuterol while they may show response to short-acting anticholinergic agents such as ipratropium bromide [24]. It is important to note that our protocol in this study was more aggressive that the one used in the usual clinical settings as we measured responsiveness to two bronchodilators with different mechanisms of action; albuterol and ipratropium bromide. We also sought to maximize the potential bronchodilator response in this study using both agents in higher than standard dosing. Pre- and post-bronchodilator spirometry was performed prior to and after inhalation of ipratropium 80 mcg followed 60 minutes later by albuterol 400 mcg. Post-bronchodilator spirometry was performed 30 minutes after inhalation of albuterol (90 minutes after ipratropium); this tended to optimize the timing to coincide with the expected peak action of each of these short-acting bronchodilators. This technique has not been utilized by any of the previously published studies evaluating BDR in COPD.

One limitation to our study is that we could only accurately measure serial acute bronchodilator responses in the placebo group due to the prolonged half-life of tiotropium, which would require a washout over several weeks. Such a washout was not feasible within the context of the UPLIFT study. Nevertheless, the placebo group still provided a large subcohort of patients in whom serial bronchodilator responses could be measured. Another limitation of long-term intervention studies in COPD is the number of prematurely discontinued patients who do not have spirometry, exacerbation, or SGRQ measurements after discontinuation from the study. However, one of the strengths of the UPLIFT study is the size and duration of the study that still provides substantial data. Additionally, while not fully compensating, statistical techniques used in the analysis do consider the issue of premature discontinuation. Finally, spirometry was not measured after albuterol alone, which limits the comparison to previous studies; however, a more important issue is the possible impact of responsiveness on changes in lung function after pharmacologic intervention. Therefore, the study is unique and provides a more comprehensive understanding of the achievable lung function improvements and the implications for therapy.

## Conclusion

In summary, the 4-year data from the UPLIFT trial demonstrate that the majority of patients with COPD had a variable occurrence of exceeding pre-defined thresholds of acute responses to short-acting bronchodilators when tested repeatedly over 4 years and only a small minority (<20%) failed to show a significant response on at least one occasion according to any threshold criterion. Furthermore, treatment with tiotropium improved lung function, improved health-related quality of life, and reduced exacerbations in COPD patients irrespective of their baseline acute bronchodilator response (BDR) and irrespective of the threshold criterion used for defining responsiveness. These findings indicate that acute bronchodilator responsiveness testing as measured in this study should not be used in predicting long term health outcomes and response to tiotropium in patients with COPD.

## Competing interests

Dr Hanania has the following relationships with GlaxoSmithKline, Dey, Sepracor, Novartis, Boehringer Ingelheim, Pfizer, and AstraZeneca: consultant, member of speakers' bureau, and recipient of research grants. Dr Sharafkhaneh has the following relationships with GlaxoSmithKline, Boehringer Ingelheim, Pfizer, and Dey: speaker, and with GlaxoSmithKline, Pfizer, and Dey: advisory boards. Drs Celli, Decramer, and Tashkin, have the following relationships with Boehringer Ingelheim and Pfizer: consultant, member of speakers' bureau, and recipient of research grants. Drs Lystig, and Kesten are employees of Boehringer Ingelheim.

## Authors' contributions

The authors meet criteria for authorship as recommended by the International Committee of Medical Journal Editors (ICMJE) and were fully responsible for all content and editorial decisions, and were involved at all stages of the manuscript's development. The authors received no compensation related to the development of the manuscript. All authors read an approved the final draft.
